# 

**Published:** 1904-09-10

**Authors:** 


### NOTICE TO CORRESPONDENTS.

All MSS., Letters, Books for Review, and other matters intended for the Editor should be addressed The Editor, "The Hospital," The Hospital Buildings, 28 & 29 Southampton Street, Strand, W.C.

The Editor cannot undertake to return rejected MS., even when accompanied by stamped directed envelope.

**Notice to Advertisers and Subscribers.**

All Advertisements, Orders for Copies of The Hospital, and Business Communications should be addressed to **THE MANAGER (Not to the Editor),** The Hospital, 28 & 29 Southampton Street, Strand, London, W.C.

The Cost of Subscription, post free, is as follows:—Per week, 3½d.; per quarter, 3s. 3d.; per half-year, 6s. 6d.; per annum, 13s. Foreign and Colonial:—Per annum, 19s. 6d., payable, in all cases, in advance.

**NOTICE.—Vol. XXXV. of "The Hospital" (October 1903 to March 1904), in handsome cloth binding, is now on sale at the office of this Journal, price 10s. 6d. Cases for binding the Numbers contained in this Volume 2s. Index to Vol. XXXV., 3½d., post free.**

**The Monthly part for September is now ready. Price 1s., by post, 1s. 3d.**

### Medical Appointments.

Sydney H. Allen, F.R.C.S.E., Medical Superintendent of the Queen Victoria Memorial Infectious Diseases Hospital, Melbourne. Richard D. Attwood, M.R.C.S., L.R.C.P.Lond., Second Assistant Medical Officer at the Southwark Infirmary. Eleanor E. Bourne, M.B., Ch.M.Syd., Resident Surgeon at the Hospital for Sick Children, Brisbane. W. B. Broster, M.B., C.M.Edin., Medical Officer to the White Lead Works, Burry Port.

St. Thomas's Hospital.—The following gentlemen have been selected as House Officers from Tuesday, September 6th, 1904:—Resident House Physicians: B. Higham, M.R.C.S., L.R.C.P.; W. Haward, M.B., B.S.Durh., M.R.C.S., L.R.C.P.; H. C. Lecky, M.A., M.B., B.Ch.Oxon. (*Extension*); C. H. Latham, M.R.C.S., L.R.C.P. (*Extension*). House Physicians to Out-patients: A. G. Gibson, B.A., M.B., B.Ch.Oxon., B.Sc.Lond.; K. Takaki, M.R.C.S., L.R.C.P. Resident House Surgeons: H. S. Bennett, M.R.C.S., L.R.C.P.; N. C. Carver, B.A., B.C.Cantab., M.R.C.S., L.R.C.P.; A. C. Birt, M.R.C.S, L.R.C.P.; G. T. Birks, M.A., M.B., B.C.Cantab. House Surgeons to Out-patients: H. A. Kisch, M.R.C.S., L.R.C.P.; G. R. Footner, B.A.Cantab., M.R.C.S., L.R.C.P.; R. E. G. Gray, M.A.Cantab., M.R.C.S., L.R.C.P.; J. C. F. D. Vaughan, M.R.C.S., L.R.C.P. Obstetric House Physicians: J. P. Hedley, M.A., M.B., B.C.Cantab., M.R.C.S., L.R.C.P. (*Senior*); H. I. Pinches, M.A., M.B., B.C.Cantab., M.R.C.S, L.R.C.P. (*Junior*). Ophthalmic House Surgeon: H. S. Stannus, M.B.Lond., M.R.C.S., L.R.C.P. (*Senior*). Special Departments: (Throat) T. B. Henderson, M.A., M.B., B.Ch.Oxon.; R. E. Whitting, B.A., B.C.Cantab. (Skin) W. L. Harnett, M.A., M.B., B.C.Cantab., M.R.C.S., L.R.C.P.; F. M. Bulley, B.A.Cantab., M.R.C.S., L.R.C.P. (Ear) T. Guthrie, M.A., M.B., B.C.Cantab., M.R.C.S., L.R.C.P.

**"The Hospital" Institutional Library.**

"Hospitals and Asylums of the World." 4 Vols., with a Portfolio of Plans, complete £12 12s.
"Burdett's Hospitals and Charities, 1904." 5s. net.
"Burdett's Official Nursing Directory." 8s. net.
"Cottage Hospitals, General, Fever, and Convalescent." 10s. 6d.
"Uniform System of Accounts for Hospitals and Public Institutions." New Edition. 4s. net.
"Hospital Expenditure: The Commissariat." 2s. 6d.
"A Legal Handbook for the Use of Hospital Authorities." 2s. 6d.
"The Cottage Hospital Case Book or Register of Patients." 10s and 12s. 6d.

**All these are published by the Scientific Press, Ltd., and may be obtained through any bookseller, or direct from the publishers, 28 and 29 Southampton Street, Strand, London, W.C.**

### A LIBRARY OF NURSING TEXT-BOOKS.

### "The Burdett Series."

Complete **10/-** post free, or separately **1/-** each, post free.

This Series comprises the undermentioned useful works, forming in itself a Complete Nursing Library. Each volume contains about 100 pp., written by a competent authority on the subject treated. They are uniform in size ($6\frac{3}{4}'' \times 4\frac{1}{4}''$), with one exception, and all are well bound in cloth boards.

Complete **10/-** post free, or separately **1/-** each, post free.

**1. PRACTICAL HINTS ON DISTRICT NURSING.**
By Miss Amy Hughes.

**2. HOSPITAL AND PRIVATE NURSING.**
By Miss S. E. Orme.

**3. THE MIDWIVES' POCKET BOOK** (with Prefatory Chapter on the Midwives Bill).
By Miss Honnor Morten, Author of "The Nurse's Dictionary," &c. &c.

**4. FEVERS & INFECTIOUS DISEASES: their Nursing and Practical Management.**
By William Harding, M.D.Ed., M.R.C.P.Lond., Author of "Mental Nursing," &c.

**5. NOTES ON PHARMACY AND DISPENSING FOR NURSES.**
New and Revised Edition.
By C. J. S. Thompson.

**7. THE CARE OF CONSUMPTIVES.**
By W. H. Daw, M.R.C.S., L.R.C.P.Lond.

**8. HOME NURSING OF SICK CHILDREN.**

By J. D. E. Mortimer, M.B., F.R.C.S.

**9. MENTAL NURSING.**
By William Harding, M.D.Ed., M.R.C.P.Lond.

**10. SICK NURSING AT HOME.**

By Miss L. G. Moberly.

**11. GYNÆCOLOGICAL NURSING.**
By G. A. Hawkins-Ambler, F.R.C.S., Author of "The Commonwealth of the Body."

**12. SYLLABUS OF LECTURES TO NURSES.**

By Andrew Davidson, M.D.

SEND POSTCARD FOR OUR NEW CATALOGUE OF STANDARD NURSING MANUALS.

*Publishers and Booksellers of all Description of Nursing Literature.*

**THE SCIENTIFIC PRESS, LTD.,** 28 & 29 Southampton St., Strand, London, W.C.

